# Combined complement and coagulation activation in ST-elevation myocardial infarction: associations with myocardial injury and dysfunction

**DOI:** 10.3389/fimmu.2025.1613603

**Published:** 2025-11-04

**Authors:** Karsten E. Kluge, Sigrun Halvorsen, Geir Ø. Andersen, Charlotte H. Hansen, Ingebjørg Seljeflot, Theis Tønnessen, Ida G. Lunde, Ragnhild Helseth

**Affiliations:** ^1^ Oslo Center for Clinical Heart Research, Department of Cardiology, Oslo University Hospital Ullevål, Oslo, Norway; ^2^ University of Oslo, Oslo, Norway; ^3^ Department of Cardiothoracic Surgery, Oslo University Hospital, Oslo, Norway; ^4^ Kristian Gerhard Jebsen Center for Cardiac Biomarkers, University of Oslo, Oslo, Norway

**Keywords:** complement system, coagulation system, STEMI, coronary artery disease, revascularization, heart failure

## Abstract

**Introduction:**

Preclinical data indicates reciprocal activation of the complement system and the coagulation cascade. The magnitude of this interaction in patients with acute myocardial infarction is unknown. We aimed to determine associations between circulating markers of complement and coagulation activation in patients with acute ST-elevation myocardial infarction (STEMI), and explore a possible link to myocardial injury and left ventricular dysfunction.

**Materials and methods:**

We included 864 patients with STEMI. Blood was drawn at a median of 18 hours after percutaneous coronary intervention. Complement activation was assessed by the terminal complement complex (TCC), and coagulation activation by prothrombin fragment 1 + 2 (F1 + 2), D-dimer and endogenous thrombin potential (ETP). Myocardial injury was estimated by peak troponin T (TnT), and left ventricular function was quantified on echocardiography by left ventricular ejection fraction (LVEF).

**Results:**

TCC was weakly correlated to F1 + 2 (r=0.086, p=0.012), D-dimer (r=0.176, p<0.001) and ETP (r=0.144, p<0.001). In multivariate binary logistic regression, there was no significant interaction between TCC and the coagulation markers on the risk of having high peak TnT or low LVEF.

**Conclusion:**

In this STEMI cohort, complement activation as measured by TCC was weakly associated with markers of coagulation activation, but the measured markers had no combined relation with the risk of high peak TnT or low LVEF. These findings suggest that while simultaneous activation of complement and coagulation cannot be ruled out, combined high levels of TCC and coagulation markers do not mirror the extent of myocardial injury or dysfunction in STEMI.

## 1 Introduction

The complement and coagulation systems are cascade systems consisting of inactive precursor proteins, rapidly activated upon stimuli (1, 2). The complement system is activated in response to invading pathogens or cellular damage, and its primary function is to mediate host defense (3). The coagulation system is activated by vascular injury, and mediates hemostasis (4). Beyond their canonical functions, both systems are involved in the pathogenesis of coronary atherosclerosis and ST-elevation myocardial infarction (STEMI) (5, 6). The complement system contributes to atherosclerosis by inducing endothelial dysfunction and triggering inflammatory signaling within the atherosclerotic plaque (7). In STEMI, the complement system contributes to plaque destabilization and ischemia-reperfusion (IR) injury (8, 9). The coagulation system contributes to atherosclerosis by triggering inflammation within the atherosclerotic plaque, as well as contributing to atherothrombosis following plaque rupture or erosion (10, 11). Atherothrombosis might cause STEMI, in which case coagulation contributes to IR injury (12), but atherothrombosis might also remain asymptomatic, where it functions as an important mechanism of plaque growth (13).

Reciprocal activation between the complement system and the coagulation cascade has been reported (2). The anaphylatoxins C3a and C5a, as well as the terminal complement complex (TCC) has been reported to activate platelets and cause them to shed pro-coagulant microparticles (MPs), and serine proteases of the complement system has been reported to activate coagulation factors (14). Conversely, coagulation factors have been reported to activate complement components directly (15), and platelets and MPs might function as platforms for complement activation (16). As a marker of “total” complement activation, TCC is well established (17). We have previously reported associations between both complement and coagulation activation to higher peak troponin T (TnT) and lower left ventricular ejection fraction (LVEF) in the present STEMI cohort (18, 19). In patients with chronic coronary syndrome, we have previously observed a link between TCC and markers of hypercoagulability (20). Associations between TCC and coagulation activation have not been reported in STEMI, where reciprocal activation could cause a self-enforcing cycle of inflammation and coagulation, theoretically resulting in a more rigid coronary thrombus and exacerbated IR injury. On this basis, we aimed to determine the associations between TCC and coagulation activation in patients with STEMI, and explore any combined relation to myocardial injury by peak troponin T (TnT) and myocardial dysfunction by left ventricular ejection fraction (LVEF). We hypothesized that TCC and the coagulation markers would be simultaneously increased, and that combined high levels would be associated with markers of myocardial injury and dysfunction.

## 2 Materials and methods

### 2.1 Study population

The study design and population have been described previously (21). In brief, patients admitted to Oslo University Hospital Ullevål with STEMI treated with percutaneous coronary intervention (PCI) were included (n=1028). Patients below 18 years of age and patients unable or unwilling to give written informed consent were excluded. Clinical information was collected from hospital records and questionnaires obtained at inclusion.

For this sub-study, patients using oral anticoagulation were excluded due to its effect on the coagulation cascade (22). A total of 164 patients were excluded, leaving 864 for the final analysis. The study was approved by the Regional Ethics Committee of South East Norway (project ID: 107832).

### 2.2 Blood sampling

Blood samples were collected in a fasting condition, between 8:00 and 10:00 a.m. the morning after PCI. For patients admitted during the weekend, inclusion was performed the following Monday morning. Blood collection was performed at a median time of 24 h after symptom debut and 18 h after PCI. Serum was prepared by centrifugation for 10 min at 2000 x g, and EDTA plasma and citrated plasma kept on ice, and prepared within one hour by centrifugation for 30 min at 3000 x g at 4 °C. Samples were aliquoted and stored at -80 °C until analyzed. All reported markers were measured in samples collected at the same time.

### 2.3 Laboratory methods

Peak TnT was defined as the highest measurement during hospitalization. The analysis was performed in serum by electrochemiluminescence technology (3rd generation cTroponinT, Elecsys 2010, Roche, Mannheim, Germany). The inter-assay coefficient of variation (CV) was 7%. Complement activation was measured in EDTA plasma by the TCC, using a commercially available enzyme-linked immunosorbent assay (ELISA) (human TCC, HycultBiotech, Uden, The Netherlands). The inter-assay CV was 8.1%. Coagulation activation was measured in citrated plasma by F1 + 2 and D-dimer, both analyzed by ELISA (Enzygnost F1 + 2; Siemens, Marburg, Germany and Asserachrom D-dimer; Stago Diagnostica, Ansiere, France, respectively), and by the endogenous thrombin potential (ETP), analyzed by the calibrated automated thrombogram assay. Inter-assay CVs were 5.4% for F1 + 2, 6.5% for D-dimer and 6.0% for ETP.

### 2.4 Echocardiography

LVEF was assessed by echocardiography within three months after the index infarction, by either visual approximation or the Simpson’s biplane method.

### 2.5 Definitions

STEMI was defined as electrocardiographic ST segment elevation of >2mm in two or more contiguous chest leads, >1mm in two or more limb leads, or new onset of left bundle-branch block, together with chest pain or other typical symptoms and troponin levels above the 99^th^ percentile (23). Previous cardiovascular disease (CVD) was defined as previous myocardial infarction, ischemic stroke, PCI procedure, or coronary artery bypass surgery. Diabetes mellitus and hypertension were defined as treated diabetes and hypertension. Smoking was defined as current smoking or cessation <3 months prior to study inclusion.

### 2.6 Statistical analysis

Data is presented as mean ± SD, median (25th, 75th percentile), or numbers (%) as appropriate. The unpaired Student t-test and Mann-Whitney U test were used to test differences between groups. Proportional data was compared using the chi-squared test. Correlation analyses were performed using Spearman’s rho. Multivariate binary logistic regression was used to quantify the individual contributions of variables, and their interactions, on risk of an outcome. P-values of ≤0.05 were considered statistically significant, and all statistical analyses were performed using IBM SPSS statistics v.27. Figures were created using Microsoft Excel v.16.x and STATA v.18.

#### 2.6.1 Statistical analysis plan

We aimed to explore associations between TCC and markers of coagulation activation by correlation analysis. To assess the magnitude of the correlation, we compared the distributions of coagulation markers dichotomized at median TCC, using a non-parametric test (Mann-Whitney U test). We prospectively combined TCC and the coagulation markers in binary logistic regression to assess possible interactions. For this analysis, above-median peak TnT and LVEF ≤40% were used as outcomes, and TCC and coagulation markers were divided into quartiles and treated as continuous variables. LVEF ≤40% was chosen as a cut-off because this is the echocardiographic criterion for heart failure with reduced ejection fraction (24). Age and gender were included in the model due to reported associations to complement activation (25).

## 3 Results

### 3.1 Study population

The characteristics of the study population are described in **Table 1**. The mean age was 61 years, 20% were female, 23% had previously diagnosed CVD, and 11% received prehospital thrombolysis.

**Table 1:** Characteristics of the study population.

Characteristic	Total study population (n=864)
Age, years, (range)	60.7 (24-94)
Female gender, n (%)	173 (20)
Smoking, n (%)	414 (47.9)
Hypertension, n (%)	283 (32.8)
Diabetes, n (%)	105 (12.2)
BMI, kg/m^2^	26.6 (24.3, 29.3)
Previous CVD, n (%)	200 (23.1)
Total leukocyte count x 10^9^/L	10.6 (8.65, 13.10)
Platelet count x 10^9^/L	219 (187, 264)
Total cholesterol, mmol/L	4.93 ± 1.94
LDL-cholesterol, mmol/L	3.25 ± 1.02
HDL-cholesterol, mmol/L	1.12 ± 0.40
Triglycerides, mmol/L	1.45 ± 0.88
Fasting glucose, mmol/L	5.8 (5.2, 6.6)
C-reactive protein, mg/L	13.39 (7.00, 31.22)
Prehospital thrombolysis, n (%)	96 (11.1)
Peak TnT, ng/L_1_	3835 (1685, 7045)
NT-proBNP, ng/L	31 (10, 116)
LVEF ≤ 40%_2_	133 (15.4)

_1_Peak TnT was defined as the highest TnT recorded during hospital stay. _2_LVEF was measured within three months of STEMI. Values are given in mean ± SD, median (25^th^ quartile, 75^th^ quartile) or number (%), unless otherwise stated. BMI, body mass index; CVD, cardiovascular disease; LDL, low-density lipoprotein; HDL, high-density lipoprotein; TnT, troponin T; LVEF, left ventricular ejection fraction; F1 + 2, prothrombin fragment 1 + 2; ETP, endogenous thrombin potential.

### 3.2 Associations between complement and coagulation markers

Statistically significant, but weak associations were observed between TCC and markers of coagulation activation (**Table 2**). When dichotomizing TCC at the median level (3200 AU), the group with above-median (“high”) TCC had significantly higher D-dimer (p<0.001) and ETP (p=0.002) levels, but no difference in F1 + 2 levels (p=0.106) (**Supplementary Figure S1**).

**Table 2:** Correlations (Spearman`s Rho) between TCC and markers of hypercoagulability.

	F1 + 2	D-dimer	ETP
TCC	r=0.086 **p=0.012**	r=0.176 **p<0.001**	r=0.144 **p<0.001**

TCC, Terminal complement complex; F1 + 2, Prothrombin fragment 1 + 2; ETP, Endogenous thrombin potential.Bold values indicate a p-value ≤ 0.05.

### 3.3 Associations between combined high complement and coagulation activation and peak TnT and LVEF

**Figure 1:**
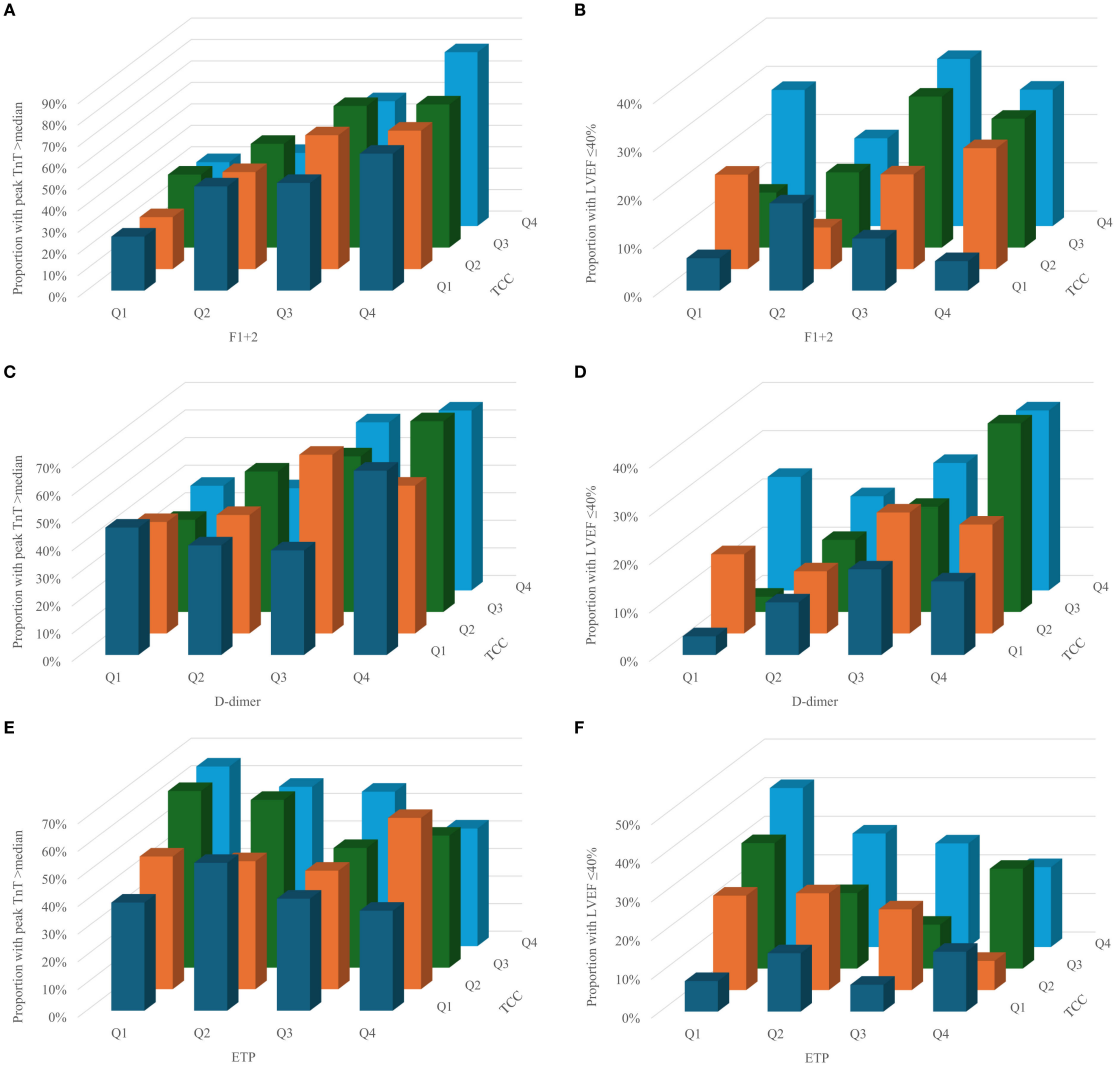
Proportion of patients with high peak TnT and LVEF ≤40% according to quartiles of TCC and F1+2 (**a** and **b**), d-dimer (**c** and **d**) and ETP (**e** and **f**).

The proportion of patients with high peak TnT and LVEF ≤40% according to quartiles of TCC, F1 + 2, D-dimer and ETP are given in **Figure 1**. Visually, there seemed to be some interaction between complement and coagulation, especially for TCC and F1 + 2 and the risk of high peak TnT (**Figure 1a**), and for TCC and D-dimer and LVEF ≤40% (**Figure 1d**). However, in multivariate binary logistic regression adjusting for age and gender, none of these were significant (**Table 3**).

**Table 3:** Logistic regression of the predictive value of age, gender, TCC and the coagulation markers on peak TnT and LVEF.

Variable	High peak TnT			LVEF ≤40%		
	OR	95% CI	p-value	OR	95% CI	p-value
TCC (per quartile)	0.903	0.655 – 1.245	0.533	1.300	0.823 – 2.054	0.260
F1 + 2 (per quartile)	1.550	1.125 – 2.136	**0.007**	1.034	0.639 – 1.674	0.892
Interaction	1.074	0.954 – 1.209	0.236	1.023	0.871 – 1.203	0.779
Age	0.993	0.981 – 1.005	0.265	1.032	1.014 – 1.051	**<0.001**
Male gender	1.329	0.924 – 1.913	0.125	1.288	0.775 – 2.141	0.329
TCC (per quartile)	0.906	0.662 – 1.239	0.536	1.193	0.700 – 2.035	0.516
D-Dimer (per quartile)	1.316	0.970 – 1.785	0.078	1.279	0.776 – 2.108	0.335
Interaction	1.059	0.946 – 1.185	0.319	1.035	0.870 – 1.233	0.696
Age	0.992	0.980 – 1.005	0.237	1.024	1.005 – 1.043	**0.012**
Male gender	1.271	0.893 – 1.810	0.183	1.379	0.825 – 2.306	0.220
TCC (per quartile)	1.484	1.096 – 2.011	0.011	1.903	1.235 – 2.931	**0.004**
ETP (per quartile)	1.139	0.836 – 1.551	0.410	1.195	0.724 – 1.970	0.486
Interaction	0.903	0.809 – 1.009	0.071	0.883	0.748 – 1.042	0.140
Age	1.006	0.994 – 1.018	0.315	1.032	1.013 – 1.050	**<0.001**
Male gender	1.151	0.807 – 1.642	0.438	1.268	0.759 – 2.120	0.364

TCC, terminal complement complex; F1 + 2, prothrombin fragment 1 + 2; ETP, endogenous thrombin potential; TnT, troponin T; LVEF, left ventricular ejection fraction.Bold values indicate a p-value ≤ 0.05.

## 4 Discussion

In this large STEMI cohort, we show that complement activation, measured by TCC, was weakly associated with coagulation activation in peripheral blood taken at a median of 18 hours after PCI. There was no significant interaction between the complement and coagulation markers and the risk of high peak TnT or low LVEF.

The participation of complement and coagulation as two distinct processes in patients with STEMI is well-established. Circulating TCC has been reported to be elevated in myocardial infarction, and cell-bound TCC is more heavily deposited in revascularized than non-revascularized myocardium (26, 27). Still, complement inhibition in acute myocardial infarction has so far mediated neutral results (28, 29). As for coagulation, F1 + 2 and D-dimer have been reported to associate with infarct size and mortality in STEMI (30–33). The literature has suggested mutual activation of the two systems through multiple mechanisms, including complement-mediated activation of platelets and direct activation of the complement system by proteins of the coagulation cascade (2).

In this first study on the clinical relevance of combined complement and coagulation activation in STEMI, we found complement activation by TCC to be weakly correlated to the coagulation markers F1 + 2, D-dimer and ETP. Despite two out of three markers of coagulation being significantly higher in patients with high levels of TCC, the associations between complement and coagulation activation were generally weak. This indicates that if there was reciprocal activation between complement and coagulation, it was of modest strength. Both the inflammatory response and coagulation activation have been shown to reflect infarct size (19, 26, 32–34), and we find it likely that other factors, such as the myocardial infarction, PCI procedure, and inflammation following ischemia and reperfusion, affected the respective activation of complement and coagulation in this very acute situation. Unfortunately, the cross-sectional study design did not permit dissection of pathophysiological mechanisms involved in potential interactions between complement and coagulation.

Based on the reported contribution of complement and coagulation on IR injury (6, 35), we hypothesized that activation of both systems would have a negative combined effect on myocardial injury and dysfunction despite the weak association between the systems. We have previously reported that the coagulation markers F1 + 2 and D-dimer were associated with peak TnT and LVEF in this population (19). Combining the complement marker TCC with the coagulation showed no interaction between TCC and F1 + 2 and D-dimer in predicting the risk of high peak TnT or LVEF ≤40%. The interaction between TCC and ETP seemed to be associated with a *lower* risk of high peak TnT. Although this could indicate that ETP modulates the effect of increasing TCC, it should be interpreted with caution, as the p-value is close to the significance value, which likely makes it a result of multiple comparisons. Taken together, these findings suggest that simultaneous activation of the two systems does not potentiate adverse clinical outcomes in STEMI. This means that despite proposed individual contributions to IR injury, these contributions do not seem to potentiate each other, and breaking this interaction might thus not be a viable pharmacological target.

### 4.1 Limitations

The cross-sectional design of this study does not permit conclusions regarding causality. A greater infarct size might lead to increased complement and coagulation activation as part of the inflammatory response during myocardial injury and heart failure development – and the proposed mechanism might be the other way around. Another limitation is that markers of complement and coagulation activation were only measured once, and their full interaction is probably not accounted for in the singe blood sample analyzed for this study. Moreover, the variable timing of blood samples after PCI should be highlighted, as time from PCI to blood sampling was correlated to the majority of markers (**Supplementary Table S1**), and the markers have been reported to peak at differing time points following myocardial infarction (36–40). There might also be differing effects depending on which complement activation pathways are activated, and this is naturally not captured when measuring only TCC. Additionally, as patients received standard pre- and in-hospital antithrombotic medication, this probably affected measured markers, as there are reports of acetylsalicylic acid, thrombolytic agents and heparins affecting complement and coagulation activation (41–48). Also, measuring circulating TCC does not account for cell-bound TCC, and the result might thus not reflect “total complement activation” (49). Finally, as neither time nor method for measuring LVEF was standardized, the LVEF results should be interpreted with caution.

## 5 Conclusions

In this large STEMI cohort, there were weak associations between circulating markers of complement and coagulation activation. There was no combined effect of complement and coagulation activation on the risk of high peak TnT or low LVEF.

## Data Availability

The data analyzed in this study is subject to the following licenses/restrictions: The dataset used during the current study is not available publicly due to Norwegian legislation about general data protection regulations, but are available from the corresponding author on request. Requests to access these datasets should be directed to Karsten Engseth Kluge, Karstenek@gmail.com.
