# Supplementary material for: Combined complement and coagulation activation in ST-elevation myocardial infarction: associations with myocardial injury and dysfunction
Source: Front Immunol. 2025 Nov 4;16:1613603. doi: 10.3389/fimmu.2025.1613603 (PMC12623171; doi:10.3389/fimmu.2025.1613603)
Supplement: Supplementary file 1 [file Table1.docx]

Supplementary Material

**Supplementary Table 1.** Correlations (Spearman`s Rho) between markers of hypercoagulability and markers associated with TCC

|  | TnT peak | NT-proBNP | CRP | Time from symptoms to PCI | Time from PCI to blood sampling |
| --- | --- | --- | --- | --- | --- |
| TCC | **r=0.070, p=0.039** | **r=0.168, p<0.001** | **r=0.311, p<0.001** | **r=0.109, p=0.002** | **r=0.266, p=<0.001** |
| F1+2 | **r=0.362, p<0.001** | **r=0.143, p<0.001** | **r=0.170, p<0.001** | r=0.003, p=0.941 | **r=0.152, p<0.001** |
| D-dimer | **r=0.258, p<0.001** | **r=0.250, p<0.001** | **r=0.339, p<0.001** | **r=0.156, p<0.001** | **r=0.121, p<0.001** |
| ETP | **r=-0.081, p=0.013** | **r=-0.106, p=0.001** | **r=0.070, p=0.033** | **r=-0.069, p=0.043** | r=-0.027, p=0.435 |
