# Supplementary figures and images for: Combined complement and coagulation activation in ST-elevation myocardial infarction: associations with myocardial injury and dysfunction
Source: Front Immunol. 2025 Nov 4;16:1613603. doi: 10.3389/fimmu.2025.1613603 (PMC12623171; doi:10.3389/fimmu.2025.1613603)

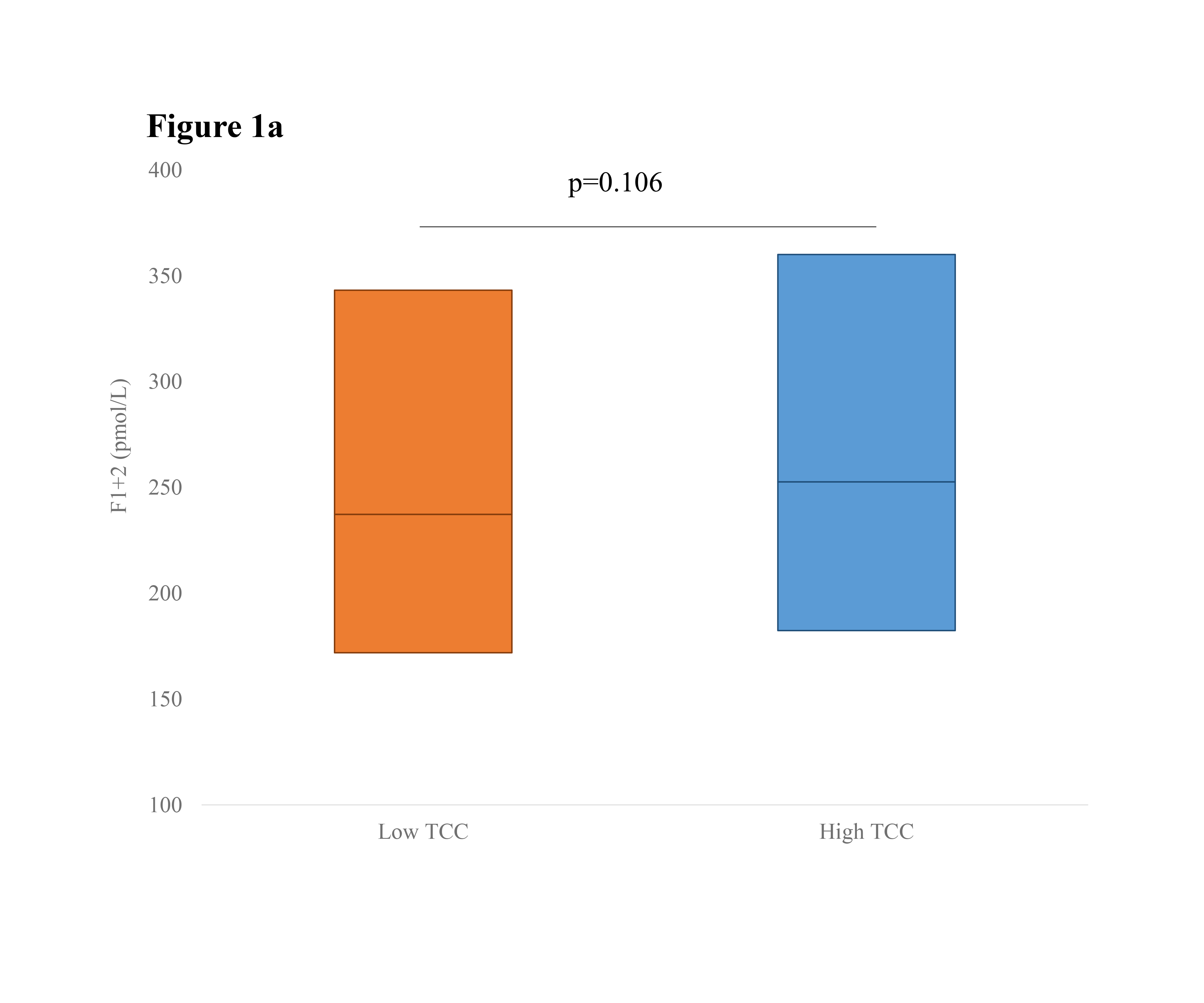

Supplement: Supplementary file 2 [file Image1.tif]

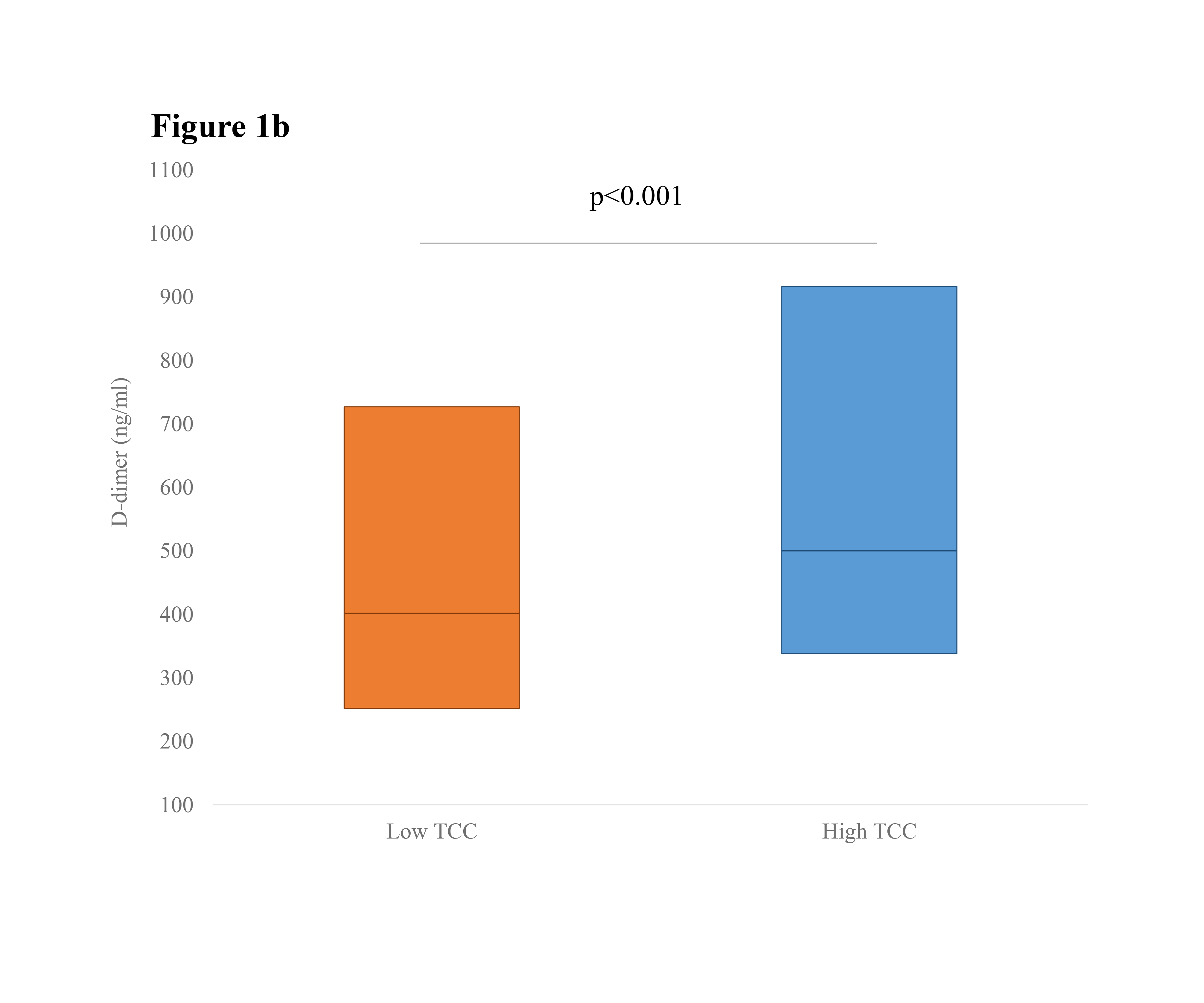

Supplement: Supplementary file 3 [file Image2.tif]

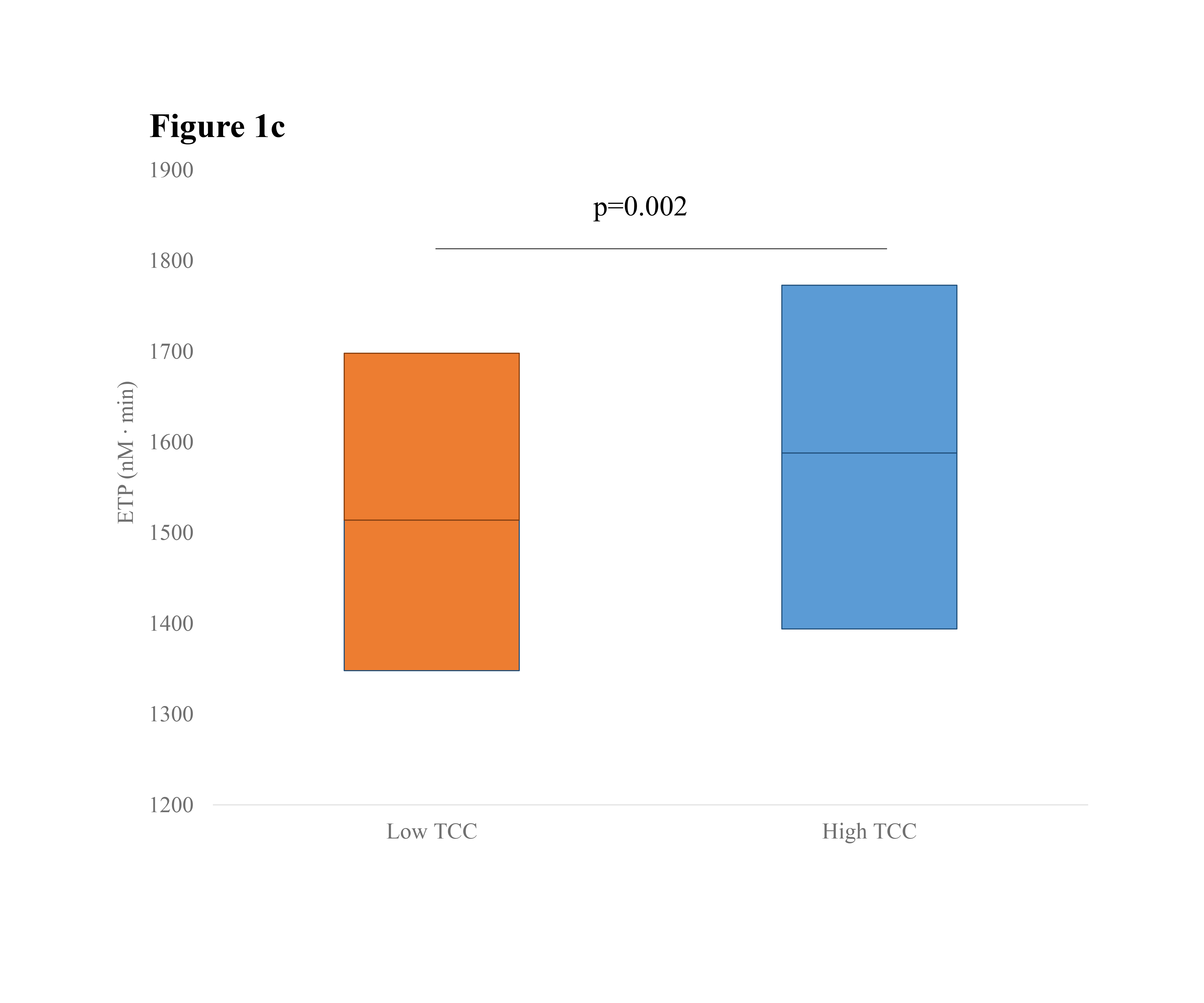

Supplement: Supplementary file 4 [file Image3.tif]
